# Perception study of state support programs for companies, in Talca, Chile in times of Covid 19

**DOI:** 10.1371/journal.pone.0274051

**Published:** 2022-10-07

**Authors:** Miguel Bustamante-Ubilla, Roberto M. Campos-Troncoso, Orly Carvache-Franco, Mauricio Carvache-Franco, Wilmer Carvache-Franco

**Affiliations:** 1 Facultad de Economía y Negocios, Universidad de Talca, Talca, Chile; 2 Sistema de Posgrado, Universidad Católica de Santiago de Guayaquil, Guayaquil, Ecuador; 3 Escuela de Auditoría e Ingeniería en Control de Gestión, CIEC, Facultad de Economía y Negocios, Universidad de Talca, Talca, Chile; 4 Universidad Católica de Santiago de Guayaquil, Facultad de Especialidades Empresariales, Guayaquil, Ecuador; 5 Universidad Espíritu Santo-Ecuador, Samborondón, Ecuador; 6 Escuela Superior Politécnica del Litoral, ESPOL, Facultad de Ciencias Sociales y Humanísticas, Guayaquil, Ecuador; The University of the West Indies, TRINIDAD AND TOBAGO

## Abstract

This work identifies the factors that influence the perception of company managers regarding the state support programs carried out in times of Covid19. A questionnaire was applied to a sample of company executives from the city of Talca, Chile. Descriptive, exploratory factor analysis and structural modeling ratified by the relevant goodness of fit indices were carried out. The results confirm the existence of three factors that affect the perception of managers that include 12 significant items. It is concluded that the investment factor acts as an independent dimension in the model, the classification factor of the companies acts as a mediator and finally the competitiveness factor turns out to be the dependent dimension of the model.

## 1. Introducción

The coronavirus, as a global threat, has been spreading for two decades, first as a respiratory syndrome and now as SARCOV-2, from a probable zoonotic source, spread by direct contact. However, confirmed diagnosis by reverse transcriptase PCR makes individual management possible by applying supportive therapy and mechanical ventilation in severe cases, in addition to reducing community spread through isolation [1]. Consequently, from the governmental perspective, two analysis options are promoted, one with a citizen focus and the other oriented to organizations to achieve subsistence [2].

In times of Covid19, governments have faced the challenge of helping people and organizations in general [3]. At the citizen level, with the help of local organizations that educate about vaccination and implement safe immunological programs [4] and at the organizational level, via business continuity plans and crisis management plans, without discriminating against these companies or institutions through the relevant legal mechanisms and in contact with corporate governments [5]. Thus, for example, at the individual level, relative success was observed in India, achieving an average response rate of 59%, leaving 6% of people resisting COVID-19 vaccination. In this way, it was possible to detect the so-called "hot spots" of segments of the population that assume negative or unclear information about vaccination [4]. In this same line of analysis, a cross-sectional study, carried out in Germany, in 2021, also reached an achievement index, identifying 57.5% of people willing to be vaccinated while 12.1% were not willing to do so, evidencing in turn, that advanced age and satisfaction with life show degrees of positive association with greater adherence to vaccination [6].

Complementarily and, as already mentioned, at the level of organizations, a second approach can be seen related to government aid that can be expressed in various ways and at different depths, for example, first in the form of transfer of funds [7], followed by the fact that State organizations can grant flexibilities of a tax nature [8], with a broad economic impact, as well as collaborating in the monitoring of performance and operational supervision of risks through the timely dissemination of relevant information that collaborate with the management and then with the management of the reputational image of the organizations [9]. In this context, as already stated by Heredia and Sánchez [2], governments intervene with the aim of mitigating the adverse effects of COVID-19 on organizations [10]. One of the short-term ways has been to facilitate remote operation, the strengthening of the team and the enhancement of available human skills. And, in the longer term, anticipating a succession plan [3] as well as anticipating the foreseeable turbulence of the environment [11].

In general, companies in Latin America [12] have turned to their States for help, which has forced governments to assume collaboration by assigning facilitating mechanisms and resources [13], focusing their contributions to those who need it most [8]. This has allowed the State to use its powers in matters of fiscal policy, public spending and tax policy [14], ensuring that these can be sustained over time [15]. Of course, it is necessary and even essential that companies receive help since, as Heredia and Sánchez [2] indicate, they face various barriers that limit or prevent their survival, highlighting the contributory role of the State [13], on the one hand, to sustain employment and on the other, to enhance the productivity and permanence of organizations [2]. In short, actions induced by governments can be key to promoting behavioral change, breaking down people’s disbelief [4] and increasing adherence levels, since, as has been studied in India, identify protective degrees of statins in patients with COVID-19 [6], which in turn generates higher degrees of trust in people and among managers that promote better behaviors in organizations [10].

To contextualize, the coronavirus disease has generated serious humanitarian and socioeconomic limitations at the country level, which are contracting economies. In the case of the Caribbean, its losses accumulate between 2020 and 2021 [16], requiring the active cooperation of developed and developing countries, precisely to overcome the challenges of the pandemic [17]. In the case of Chile, during the last decade, the number of companies increased. Half of the firms are 14 years old or less, 49.1% of the organizations are constituted as natural persons and 48.3% are family businesses. In general, the sector that brings together the largest number of firms is commerce [18]. Thus, for example, for Solimano et al. [15] an important contribution to the Chilean market has come from Small and Medium Enterprises (PYMES) since they stand out for the generation of jobs and for the increase in competition in the markets, adding dynamism to them. In the same way, Cancino and La Paz [19] indicate that SMEs are fundamental in economic development insofar as they contribute, in a multiplicative way, to the increase in the potential growth capacities of the country’s internal product.

To the process described, large companies are added, which, given their size and experience, incorporate state-of-the-art technology expressed in investments in R&D, credits and financing, evidencing the structural superiority of these organizations [15]. However, although according to Ochoa et al. [20] the creation of SMEs increases periodically, a significant proportion of these fail to remain in the market for more than 5 years. Micro, Small and Medium Enterprises (MSMEs) are added along this same line, whose innovation capabilities are limited to the incorporation of information and communication technologies (ICT), since in the short term they have facilitated their processes and generated some growth spaces for its sales and greater profitability [21].

### 1.1 Government aid

In the opinion of Antunes, et al. [22] governments are obliged to execute rescue plans for companies, precisely because their timely intervention [2] would allow sustaining the production, financing and stability of the companies. jobs in times of turbulence. Thus, for example, at the level of the general population, people with economic problems depend on health benefits from State institutions, from those who receive medical care and regular check-ups, which, however, require long waiting periods to generalize their beneficial effects, which is why, at the international level, a solid strategy requires pandemic management programs at the level of the countries that make up the South American region [23]. As of July 2020, it affected Trinidad and Tobago, which has a Human Development Index (HDI) of 0.79 and which positions it as the richest Caribbean country in the area, on the contrary, the North Caribbean Island, Haiti, received the impact of COVID-19 in the month of May of the same year, deteriorating its relative position and placing it in position 170 of 189 countries in the world [24]. Similarly, the islands of Jamaica, Aruba, Bahamas and Saint Vincent suffered the effects of COVID-19 also during the year 2020, being monitored and informed weekly to achieve greater effectiveness of their public policy [23]. However, governments seek to sustain their economies [12] through subsidies and transfers [7] as well as perfecting regulations [25]. From this perspective, in Australia, the state intervened by stimulating the development of companies beyond its regulatory role [26], while in Romania, the benefits were delivered under limited conditions and terms [26].

In this context Rincón and Mujica [27], analyzed the impact produced by the Barrio Adentro ll program in the State of Zulia, Venezuela, applying the method of participatory perception seeking to relate problems with social programs, confirming the good reception that free programs have. for the benefit of the community [27]. A second study carried out in the Biobío Region, Chile, analyzed the perception of organizations on the occasion of the 2017 presidential elections, highlighting variables such as political uncertainty and decreased demand, confirming that the priority of organizations is more than anything growth followed by its alignment with the environment [28]. Finally, in Uruguay in 2019, the contingency in the country was studied, determining that Uruguayan firms perceived that economic activity, investment and employment levels would decrease, however, when comparing these perceptions with those obtained in 2018, it was possible appreciate a slight percentage increase in the positive vision of the future by Uruguayan companies [29].

### 1.2 Perception

Perception [30] is the result of a reflection process carried out by a person on the elements that make up the environment, relating stimuli to receptors and which has been ratified by Arias [31] and Seland [30] who refer to the perception to the recognition and interpretation that the individual makes of the information captured through the senses, and that Kumar [32] classifies as diverse and even opposed between different individuals. This is how Arias [31] defines perception as an endogenous human capacity, analogous to each person, confirming the findings of Bustamante-Ubilla et al. [33] when he points out that perception depends on the context that surrounds the individual and the interpretation let him make his reality. In short, perception collaborates in the construction of mental models or schemes [34] that allow the person and the entities they lead to know, understand and assume a certain reality in order to act in it in a coherent and effective manner [35].

Based on the above, the present work identifies the latent factors, the relationships and incidences that determine the perception components of the state support programs in times of Covid-19 of the managers of the companies in Talca, Chile.

## 2. Methodology

This work was carried out by applying a cross-sectional and correlational study of the data [36]. First, a descriptive analysis of the sample of managers interviewed on behalf of their respective companies was carried out. Secondly, a first inferential analysis was carried out aimed at achieving the focus of the dimensions under analysis and which was carried out by applying Exploratory Factor Analysis, AFE [37]. Finally, progress was made towards the relationship and causality analyzes of the key dimensions by applying Exploratory Structural Equation Modeling, ESEM, as a semi-confirmatory alternative [38] and which were carried out by applying analysis of covariance and variance, respectively. Finally, the data processing was carried out using the SPSS V23 software, for the descriptive analyzes [36], additionally the Factor.10.4. win64 program was used for the exploratory analysis, AFE [39] and finally for the ESEM analysis, the IBM—SPSS—Amos program was used.

To carry out this investigation, the data sources of the Department of Economic and Tax Studies of the Subdirectorate of Strategic Management and Tax Studies of the Internal Revenue Service, SII [40], were available as of July 2019 and the data from active commercial patents during the first quarter of 2020, issued by the Headquarters of the Transparency Unit of the Illustrious Municipality of Talca [41], dated April 4, 2020. From these two sources of data, a random search of cases was carried out in order to organize the interviews necessary for the study.

The database was organized in the Excel program, listing each of the available contacts after verifying the completeness of the records, proceeding to eliminate duplicate records. Subsequently, the random case search option was used to identify the organizations to be interviewed. Finally, a random list of companies to contact was obtained.

### 2.1 Population and sample

The sample unit of the investigation was the companies selected according to the inclusion criteria. As a first criterion, the study focused on those located in the city of Talca, Maule Region of Chile, formally represented by their respective directors. The second inclusion criterion was that they were formally active companies in the official records of the State, managing to identify a population of companies in the city of Talca that totals 1,375 organizations from various sectors and sizes, forming a finite universe. Naturally, as an exclusion criterion, the other companies in the region located in three cities of the region (Curicó, Linares and Cauquenes) were not included in the universe of studies. For the determination of the sample a priori, an error of 5% was foreseen, a maximum variance of 0.25, corresponding to a probability of success and failure of 50% (p = 0.5 * q = 0.5) and a confidence level of 95%, reaching a total of 301 cases.

### 2.2 Interviews

Given the context of Covid 19, it was planned to conduct the interviews [42] p.217 in this case, through the application of an online questionnaire, feasible to be implemented through the Google form website, to be sent electronically. to the contacts of the previously determined sample, incorporating a direct access link to the digital questionnaire.

The questionnaire was addressed to the directors of the companies, who are assumed to have the necessary knowledge of the activities of their respective organizations as well as the information on the necessary government aid that allows them to face the particular environments of each of the companies interviewed. Based on these minimum criteria, valid and reliable information could be collected for the study.

### 2.3 Instrument description

After a previous study of variables related to state aid available in the Chilean context, it was possible to create an initial list of 57 items, which were analyzed and regrouped by similarity, creating a refined inventory of questions to generate a questionnaire. He foresaw the consultation of a group of 7 experts, university professors and business managers, who were presented with an initial questionnaire [42] p.217 to collect their observations regarding the questions asked regarding state support programs in the contingency context caused by the corona virus pandemic (COVID—19). Once the observations were received, the duly revised instrument was structured into three sections containing the questions defined and approved to be presented to the interviewees in correlative order and associated with a five-point categorical scale [43].

Once the design of the questionnaire was completed, the instrument was revised and adjusted, verifying its text and its corresponding adaptation to the context of application [44]. Content and structure were verified by a panel of experts who evaluated the texts of each question, verifying their syntax, wording and unidirectionality of the statements in order to achieve an instrument that was easy to read and understand by the interviewees. In addition, a field validation was carried out through the application of pilot tests aimed at finding expression, writing and syntax errors from the perspective of the interviewees, to ensure the due understanding of the interviewees to the proposed items. An adequate selection of the subjects planned for the study was taken into consideration, however, ensuring that these contacts were not incorporated into the final study sample. Finally, the correction of the writing and coherence of expression and writing that were pertinent to incorporate were made. The pilot test contacted was 10% of the planned sample in order to verify the operability of the instrument [33].

The Project was approved by the ESPOL University Research Dean with Code FCSH-14-2021 and the consent of the participants was informed in writing at the beginning of the questionnaire where the participants agreed to participate in the study.

The instrument contains an introduction in which the objective of the study is disclosed. In the first part, the questionnaire collects the characteristics that allow defining the profile of the entities. In the second part, a list of statements is presented that refer to government programs of state aid to organizations in Chile, especially relevant in times of Covid 19. In the third part, a series of statements are presented regarding general aspects related to the mechanisms for assigning, distributing and obtaining resources foreseen in government programs of government support. Finally, both in the second and in the third part of the instrument, the responses of the interviewees are collected using a discrete Likert scale of 1:5 points [39].

### 2.4 Factor analysis

An exploratory factorial analysis of the observable items was performed [45] seeking to reduce the observations to a limited number of latent dimensions that synthesize the instrument [39]. For this, the data adequacy index was verified by calculating the minimum Kaiser-Meyer-Olkin coefficient (KMO) (≥ 0.6) and a significant Bartlett sphericity test (***) [46], which allows verifying that the correlation matrix is different from the identity matrix, with correlation coefficients other than 0 and looking for the value (p) to be less than the expected significance value (≤ 0.05) [47].

The extraction and rotation of factors [45] took into account the respective eigenvalue corresponding to the measure of variability of the amount of information that can explain a factor, assigning an initial value equal to 1, which allows to explain most of the total variability of the observable items under study [48] and, for the conformation of the factors, it was decided to require high communality indexes (≥ 0.3) from the reagents and factor loadings of the reactive with the factor also high (≥ 0.5) [45].

### 2.5 Structural modeling

The approach of the validation model [49] was formulated with all the reagents using the Method of Structural Equations (Exploratory Structural Equation Modeling) ESEM, as a semi-confirmatory alternative, [50]. In addition, given the response options of the questionnaire on a Likert scale 1:5 points that allow collecting frequencies that behave in an approximately normal way [46], it was possible to assume that it was possible to overcome the risks that could lead to non-linear relationships. Therefore, we worked with the observable variables or instrument items defining them as polychoric [39], which were characterized as endogenous, mediating or exogenous latent variables to design the structural equation model [50].

The previous steps of the confirmatory analysis suggested by Kaplan [48] were taken into consideration and the structural analysis was carried out to ratify the latent variables of the model [50, 51], opting for sensitize standardized values and significance indices of the estimators [38]. To ratify the findings, the Comparative Fit Index (CFI), Goodness of Fit Index (GFI) and Adjusted Goodness of Fit Index (AGFI) were observed [50], verifying that their values are between values 0 and 1, where 1 indicates a perfect fit.

Finally, the goodness-of-fit analyzes were performed considering the Chi-Square Absolute Statistical Indices-Likelihood Ratio (CMIN/DF ≤ 3) [50], the Normed Fit Indices (NFI) and the Non-Normed Index of Fit or Tucker Lewis Index (TLI) (≥ 0.9) [50], in addition to the non-Normed Index of Fit or Tucker Lewis Index TLI (≤ 0.9) [39] and the Normed Fit Index NFI (≤ 0.9) [52], ending with the Mean Square Error of Approximation Index (RMSEA) (≤ 0.05) less than the maximum required [39].

### 2.6 Use of software

The analysis and processing of the data was carried out using the statistical software SPSS V23 [36]. For modeling using structural equations, AMOS from [39, 51] was used, applying the Maximum Likelihood method, ML. Furthermore, in order to reduce the effects of variance [53], standardized estimators were interpreted.

## 3. Resultados

The results of the descriptive, factorial and structural equation modeling analyzes are presented below.

### 3.1 Descriptive analysis

The real sample of interviewees reached 313 managers of companies in the city of Talca, which allowed reducing the margin of error of the study from 5% to 4.88%.

Observing Table 1, 54.3% of the sample corresponds to small companies; while 30% corresponds to medium-sized companies. 31.3% of the companies in the city of Talca have been in the market for 9 years or less. 75.4% of the sample has been a beneficiary of state programs. These benefits have been granted more frequently by state entities such as the Technical Cooperation Service, Sercotec (15.7%), Production Development Corporation, Corfo (14.1%) and ProChile (10.5%). It can also be seen that the perception of the general aspects of government support programs are answered in the categories of agreement and totally agree, on the Likert scale equivalent to the higher frequencies (4 and 5). Regarding, if the seniority of a company determines whether or not it accesses benefits, the option of agreement adds 30.7% to which the totally agreement alternative is added, which reaches 26.6%, totaling 57.3%. In relation to whether the benefits are currently accessible to all types of companies, the totally agree option reaches 34.5%, while the agree alternative amounts to 28.1%, adding 61.6%.

**Table 1:** Perception of the general aspects of the state support programs.

Variable	Response category	Frequency	Percentage
Do you think that the age of a company determines whether or not it accesses benefits?	In agreement	96	30.7%
	Totally agree	82	26.6%
Do you think that the benefits are currently accessible to all types of companies?	Totally disagree	108	34.5%
	Disagree	88	28.1%
Do you think the benefits should be directed to regions with high unemployment rates?	In agreement	100	32.3%
	Totally agree	101	31.9%
Do you think that if the requirements for benefits are reduced, their application will increase?	In agreement	83	26.5%
	Totally agree	226	72.2%
Do you think there is a need to improve the current promotion benefits?	In agreement	106	33.9%
	Totally agree	150	47.9%

Source: The researcher. Research project: Government support for companies in Talca, Chile, in times of Covid-19.

On the other hand, when asked if the benefits should be directed to regions with high unemployment rates, the interviewees indicated that they agreed with a relative frequency of 32.3%, followed by those who totally agreed that they reach 31.9%, reaching the sum of 64.2%. In addition, regarding whether the requirements for benefits are reduced, the application for them would increase, the interviewees who answered the category of agreement marked 26.5% and those who registered the option totally agreed reached 72.2%, adding 98, 7%. Finally, regarding whether it is necessary to improve the promotion that the benefits currently have, the responses again focus on the superior response options. In agreement they marked 33.9% and totally in agreement they registered 47.9% of the interviewees, totaling 81.8%.

### 3.2 Factor analysis

The factorial analysis of principal components considering extraction by the varimax method, managed to determine a KMO index of 0.793, a significant Bartlett Sphericity test (0.000) and a significant Cronbach α reliability index of 0.752 (***) for the instrument in its entirety (Table 2).

**Table 2:** Perception factors of the state support program for companies.

Factor 1: Investment			
Items	Descriptor	Factor loading	Communality
17	Do you think that access to government benefits will allow your company to develop more efficient processes?	0.508	0.342
18	Do you think receiving training will allow your company to develop greater production at a lower cost?	0.660	0.491
19	Do you think that if your company receives money from the State, you can increase salaries?	0.801	0.655
20	Do you think that if your company receives money from the State, it could be invested in infrastructure?	0.759	0.578
21	Do you think that if your company receives money from the State, it can design new products and services?	0.758	0.577
24	Do you think that if your company receives benefits, it could improve the working conditions of its employees?	0.662	0.440
Variance Explained	25.439%	Reliability	0.801
Factor 2: Company Classification			
12	Do you think that the economic benefits should vary according to the company’s size?	0.743	0.593
13	Do you think that the economic benefits should vary according to the company’s market?	0.827	0.725
14	Do you think that the economic benefits should vary according to the company’s current financial situation?	0.702	0.506
Variance Explained	15,050%	Reliability	0.650
Factor 3: Market Competitiveness			
6	Do you think that benefits allow a company to be more competitive?	0.668	0.519
7	Do you think that if your company obtains benefits, it will consolidate itself in the market?	0.750	0.610
10	Do you think that if a company with a track record receives benefits, it increases market obstacles?	0.743	0.555
Variance Explained	14,422	Reliability	0.562
Kaiser–Meyer–Olkin coefficient, KM O	0.793	Bartlett’s Sphericity Test	0.000
Total Variance Explained	0.562	Instrument Reliability	54,912

Source: Researcher. Research project: Government support for companies in Talca, Chile, in times of Covid-19.

The factorial allowed to determine three factors that group 12 reagents with their corresponding factorial loads and communality indices. In general, the factors confirm that all the items reach higher communality indices (≥ 0.30) and that their factor loads with their respective factors are also high (≥ 0.50) (Table 2).

Based on the factors detailed in Table 2, the first factor called F1: Investment, made up of 6 items (V17, V18, V19, V20, V21 and V24) achieves an explained variance of 25.439% and a reliability index α Cronbach’s of 0.801. The second F2 called Company Classification made up of 3 items (V12, V13 and V14) explains 15.050 of the variance and reaches an α of 0.65. Finally, the third factor F3, called Market Competitiveness, contains 3 items (V6, V7 and V10) with an explained variance of 14.422% and a Cronbach’s α of 0.562, totaling 54.912% of the total variance. Consequently, it can be affirmed that the factor (F1) investment prevails over the factors classification of companies (F2) and market competitiveness (F3) in the perception that managers of Talca companies have regarding state benefits.

In a first interpretation, it can be inferred that the investment factor (F1) and its respective reagents refer to the importance for managers of the investment that can be made within the organization with the benefits obtained. According to this, this investment factor F1 is directly related to the allocation of the benefits received, which are used within the company in infrastructure, processes, products; as well as they are destined towards human resources, in the person of the employees, for example, investing in remuneration and better working conditions.

The factor called classification of companies (F2) is linked to aspects related to obtaining state benefits, of course according to the type of company. This factor corresponds to the scenario in which companies currently find themselves; It also refers to the type of company and markets to which they belong and, of course, in relation to the financial situation that distinguishes between healthy companies or those that have faced some type of failure, emphasizing the importance of classifying a company in any of these categories at the time of applying for state benefits, to be effectively beneficiaries of these.

Finally, the factor (F3) related to market competitiveness is related to the competitiveness variables, narrowing the relationship between obtaining state benefits and the increase in the company’s competitiveness in the market. From this perspective, this last factor refers to the increase in competitiveness by means of obtaining benefits, which will allow companies to establish themselves in the market and, at the same time, increase the entry barriers for new companies that intend to enter the market. the domain space of each of them in the industry.

### 3.3. Structural modeling

The results obtained from the structural modeling analyzes using the covariance and variance methods to determine the relationships and incidences that occur between the independent, mediating and dependent factors under study are described below.

#### 3.3.1 Análisis de covarianza

Through the covariance analysis and applying the maximum likelihood method, ML, it was possible to identify the relationships that occur between the determined factors and that are presented in detail in Table 3.

**Table 3:** Estimators of the covariance modeling.

Items	Estimator	I KNOW	CR	Standard estimator	P
V6	4,038	0.058	70,204	0.586	***
V7	3,818	0.055	69,746	0.705	***
V10	3,489	0.083	41,873	0.404	***
V12	4,019	0.058	69,670	0.395	***
V13	3,847	0.066	57,994	0.344	***
V14	4,498	0.038	116,889	0.592	***
V17	3,962	0.055	72,659	0.667	***
V18	3,514	0.062	56,669	0.586	***
V19	3,125	0.071	44,119	0.513	***
V20	3,834	0.064	60,189	0.571	***
V21	3,725	0.068	54,747	0.534	***
V24	3,438	0.061	56,632	0.599	***
Factor Estimators					
Factor	Estimator	I KNOW	CR	Standard estimator	P
F1	0.413	0.045	9,117	0.142	***
F2	0.162	0.040	4,032	0.074	***
F3	0.354	0.073	4,828	0.075	***
Ratio Estimators					
F2← →F1	0.024	0.026	0.917	0.092	0.049**
F2← →F3	0.052	0.027	1,934	0.218	0.053*
F3← →F1	0.174	0.037	4,744	0.456	***
Goodness-of-Fit Indicators					
Model	NPAR	CMIN	DF	P	CMIN/DF
Default	38	84,859	52	0.003	1,632
Saturated	90	0	0		
Independent	24	863,843	66	0	13,089
Model	NFI Delta 1	RFI rho1	IFI Delta 2	TLI rho2	IFC
Default	0.902	0.875	0.96	0.948	0.959
Saturated	One		one		one
Independent	0	0	0	0	0
Model	RMSEA	90’s	HI90	PCLOSE	
Default	0.045	0.027	0.062	0.666	
Independent	0.197	0.185	0.209	0	

Source: The researcher. Research project: Government support for companies in Talca, Chile, in times of Covid-19.

The relationships of the 3 extracted factors are appreciated. The covariance between the company classification factor (F2) and the investment factor (F1) is 9.2%, between the company classification factor (F2) and the market competitiveness factor (F3) is 21.18%. and, between the market competitiveness factor (F3) and the investment factor (F1) it is 45.6%, which demonstrates the potential of these relationships.

The goodness-of-fit indices of the model are confirmatory. The CMIN/DF index reaches the value 1.632 (≤ 3), CFI equal to 0.959 (≥ 0.90) and an RMSEA error index equal to 0.045 (≤ 0.05), which ratifies a correct adjustment of the data confirming the relationship model obtained through the covariances, so that it effectively allows us to assume that the three factors of the model are related in a pertinent way.

#### 3.3.2 Análisis de varianza

The analysis of variance allowed determining the incidence relations between the determined factors. In general, it was possible to determine the respective rates of effect of one factor on another, determining a dependent factor, a second mediator and an independent factor. Table 4 shows the cause-effect relationship between the factors.

**Table 4:** Variance modeling estimators.

items	Estimator	I KNOW	CR	Standard estimator	P
V6	4,038	0.058	70,210	0.344	***
V7	3,818	0.055	69,746	0.495	***
V10	3,489	0.083	41,869	0.164	***
V12	4,019	0.058	69,652	0.156	***
V13	3,847	0.066	57,968	0.118	***
V14	4,498	0.038	116,915	0.351	***
V17	3,962	0.054	72,931	0.448	***
V18	3,514	0.062	56,604	0.343	***
V19	3,125	0.072	43,513	0.256	***
V20	3,834	0.064	60,137	0.325	***
V21	3,725	0.068	54,709	0.285	***
V24	3,438	0.061	56,669	0.359	***
Factor Estimators					
Factor	Estimator	I KNOW	CR	standardized estimator	P
F1	0.412	0.045	9,125	0.009	***
F2	0.161	0.040	4,012	0.007	***
F3	0.272	0.060	4,528	0.232	***
Incidence Estimators					
f2 ←f1	0.052	0.063	0.835	0.084	0.024**
F3 ←F2	0.269	0.166	1,621	0.182	0.065*
F3 ←F1	0.400	0.084	4,782	0.432	***
Goodness-of-Fit Indicators					
Model	NPAR	CMIN	DF	P	CMIN/DF
Default	39	78,484	51	0.008	1,539
Saturated	90	0	0		
Independent	24	803,843	66	0	13,089
Model	NFI Delta 1	RFI rho1	IFI Delta 2	TLI rho2	IFC
Default	0.909	0.882	0.996	0.955	0.966
Saturated	one		one		one
Independent	0	0	0	0	0
Model	RMSEA	90’s	HI90	PCLOSE	
Default	0.042	0.022	0.059	0.77	
Independent	0.197	0.185	0.209	0	

Source: The researcher. Research project: Government support for companies in Talca, Chile, in times of Covid-19.

It is shown that the investment factor (F1) influences the company classification factor (F2) by 8.4%, while the company classification factor (F2) influences the market competitiveness factor (F3) by 18. two%; On the other hand, the investment factor (F1) has a 43.2% influence on the market competitiveness factor (F3). Checking these incidence relationships, the adjustment coefficients of the structural model present conforming results, starting with the CMIN/DF index equal to 1.539 (≤ 3) which ratifies the model adjustment, an RMSEA error index equal to 0.042 less than the maximum required (≤ 0.05) with which it confirms the approximation of the model with reality and a CFI index of 0.966 (≥ 0.9) which indicates that the model is acceptable.

**Fig 1:**
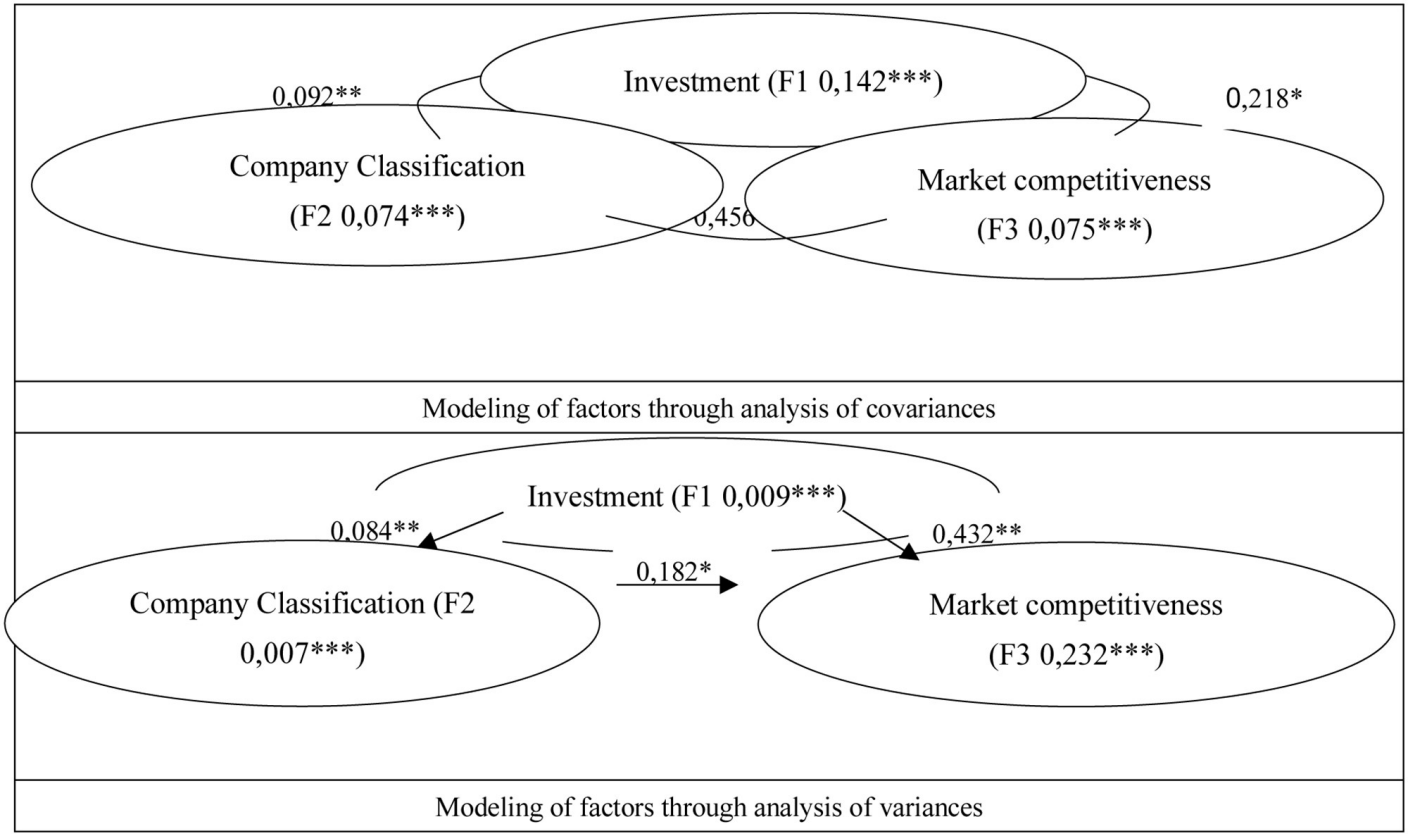
Structural modeling of covariance and variance. Source: The researcher. Research project: Government support for companies in Talca, Chile, in times of Covid-19.

In addition, Fig 1 shows the modeling of the factors according to the analysis of covariances [51, 53] that establishes the mutual relationships between the factors and also shows the model determined by the variance method that establishes the causalities between the factors [39, 50].

According to this modeling that determines incidences, the investment factor (F1) acts as the independent factor, the market competitiveness factor (F3) as the dependent factor of the model. Finally, the company classification factor F2 assumes a mediating role in the modeling.

## 4. Discusión

Starting the analysis, it is appreciated, in the first place, the relevance that the management and handling, by state agencies, of the pandemic from its origin and its progressive evolution to the present had and has to date.

In this regard, the directors interviewed, just like each of the Chileans, were subject to the plans that the State in general established for the management of COVID-19. Thus, for example, the vaccination status of the study sample evolved progressively according to the implemented plans [54]. In this context, as of December 2019, the Epidemiology Department of the Ministry of Health created the Epivigila computer platform (p.28) associated with a Preparedness and Response Plan for epidemiological surveillance, diagnostic reinforcement, and risk communication (p. 30), giving rise to a Health Alert plan for all of Chile that successively extends until the present year 2022 (p.35). In addition, the COVID-19 Advisory Council of a scientific-technical and multidisciplinary nature was created [54], which advises on the prevention, diagnosis, approach and treatment of COVID-19 (p.57) and which is accompanied by the creation of the COVID-19 Social Table, made up of government authorities, mayors, academics, and health professionals, as an instance of coordination, dialogue, and collaboration (p.65) for the implementation of public policy [54]. As a corollary, in January 2021, PAHO described the Chilean pandemic management strategy as successful, highlighting the role of the Health Residences, the Integrated Public and Private Health Network COVID-19, validating a strategy led by the Ministry of Health (Exempt Resolution 156, p.69). Finally, the fiscal effort is valued, which, by mid-2021, has 17,200 new permanent health professionals and 32,000 additional personnel to meet specific needs for critical periods [54]. Finally, by the end of 2021, 20% of the population would have been rehabilitated, whose projection by 2022 reaches 40% of the target population, and by 2023 it is expected that therapies would be delivered to 40% of the remaining population [54] p.137.

From the technical perspective of the analysis, the present job contributes to the knowledge of the factors that influence the perception of company managers regarding state benefits. Relationships can be established between the items contained in the extracted dimensions and the variables mentioned in the theoretical framework regarding State intervention.

Regarding the investment factor (F1), company directors consider investing the funds received in the company’s production processes, in addition to designing and improving the products and services offered, which according to Benedettini et al. [55] contributes to the decrease in the probability of bankruptcy.

Regarding the factor called classification of companies (F2), the directors of the firms agree with the segmentation according to the size of the company, as indicated by Crespi [56], Lopucki & Doherty [57]. Like Barboza et al. [58], the directors of the companies consider that the delivery of benefits should vary according to the current financial situation of the organization. As mentioned by Alaminos et al. [59] and Arroyave [60] the financial situation will be crucial to predict the future scenario of the firm.

Continuing with the market competitiveness factor (F3), the directors of the organizations agree with the relationship between obtaining benefits and increasing market competitiveness, a situation that, according to Heredia & Sánchez [2], makes it timely to intervention of the State towards the companies. Consequently, given that in conditions of pandemic restriction the feasibility of competing in the market is restricted and barriers are increased, adjustment processes are stimulated, as pointed out by Yoguel et al. [61], precisely to be able to adapt to changes.

## 5. Conclusiones

Regarding the objectives set and the results obtained, it is concluded that, in general, the variables that make State intervention in companies opportune in pandemic conditions turn out to be market competitiveness, financial management of organizations, level of indebtedness, financing barriers, productivity and levels of innovation.

It is ratified that the directors of the Talca firms recognize the importance of investing the benefits received within the organization to generate improvements, considering the segmentation of the firms to apply for and obtain state benefits relevant. Regarding market competitiveness, they identify the relevance of obtaining benefits for the development of a firm, which brings with it an increase in market competitiveness and the raising of barriers. Consequently, the dimensions that bring together the key components of the perception of the managers of the companies in Talca regarding the state benefits that they can access in times of Covid-19 are synthesized three factors of investment, classification of companies and market competitiveness.

Finally, among the three key factors identified, relevant relationships and incidences are established, concluding that the investment factor acts as an exogenous and independent factor, the company classification factor assumes a mediating role in the modeling and the market competitiveness factor, It turns out to be the endogenous or dependent factor within the structural model that determines the perceptions of the managers of companies in Talca, regarding state support programs in times of Covid-19.

## 6. Limitation

Although the present work managed to identify a population of 1,375 managers gradually accessible due to their location in the city of Talca, it would have been interesting to have been able to access a broader sample of the other cities of the Maule Region, such as those located in Curicó, Linares. and Cauquenes, however, a significant sample of 313 executives representative of the companies in the city of Talca was achieved, reducing the margin of error of the analyzes carried out.

As a result of the above, it is estimated that the steps of analysis, follow-up and subsequent additional studies that are suggested and that derive from the work carried out, are proposed to address the issue from three complementary dimensions. First, carry out a second study in the same city of Talca once the end of the pandemic is declared and the adverse effects of COVID-19 have been reduced. Second, to carry out a broader study of regional coverage that incorporates, on this occasion, the other cities of the Maule region of Chile, in order to obtain a broader analysis that allows ratifying the findings reached in this first work. Finally, it is proposed to validate a research instrument, in the form of a psychometrically structured questionnaire, that allows the future study of state support for organizations in times of social impact.

## Supporting information

S1 File (SAV)

S2 File (DOCX)

## References

[1] Umakanthan S, Sahu P, Ranade AV, Bukelo MM, Rao JS, Abrahao-Machado LF, et al. Origin, transmission, diagnosis, and management of coronavirus disease 2019 (COVID-19). Postgrad Med J. 2020; 96:753–758. 32563999 10.1136/postgradmedj-2020-138234 PMC10016932

[2] Heredia L, Sánchez JI. Evolution of public policies to promote SMEs in the Andean Community of Nations and the European Union: a comparative analysis*1. Revista Finan y Polít Econom. 2016; 8:221–249.

[3] CAF, Banco de desarrollo de américa latina. Visiones: Covid 19, gestión de crisis y gobierno corporativo, 07 mayo, 2020; 2020 [cited 2022 May 1]. https://www.caf.com/es/conocimiento/visiones/2020/05/covid19-gestion-de-crisis-y-gobierno-corporativo/

[4] Subramaniam N, Sharma R. COVID-19 Vaccine Hesitancy and Resistance in India Explored through a Population-Based Longitudinal Survey. Vaccines. 2021; 9:1064. doi: 10.3390/vaccines9101064 34696172 PMC8537475

[5] Rao K, Tilt C. Board Composition and Corporate Social Responsibility: The Role of Diversity, Gender, Strategy and Decision Making. J Bus Ethics. 2016; 138:327–347. doi: 10.1007/s10551-015-2613-5

[6] Umakanthan S, Senthil S, John S, Madhavan MK, Das J, Patil S, et al. The protective role of statins in COVID-19 patients: a retrospective observational study. Translatio Med Commun. 2021; 6:1–10. doi: 10.1186/s41231-021-00102-4 34604534 PMC8475829

[7] Benitz R, Ruscello C. Credits and incentives updates: Game changer or business as usual for state incentive programs. J State Tax, 2017; 35:13–55. [cited 2022 May 1]. Available from: https://search-proquest-com.utalca.idm.oclc.org/docview/1876042497?accountid=14675

[8] SII. News: Tax measures are implemented to support people and SMEs. March 19, 2020; 2020 [cited 2022 May 1]. https://www.sii.cl/noticias/2020/190320noti01srm.htm

[9] Grover P, Kar A, Ilavarasan V. Impact of corporate social responsibility on reputation—Insights from tweets on sustainable development goals by CEOs. Int J Inf Manag. 2019; 48:39–52. doi: 10.1016/j.ijinfomgt.2019.01.009

[10] He H, Harris L. The impact of Covid-19 pandemic on corporate social responsibility and marketing philosophy. J Bus Res. 2020; 116:176–182. doi: 10.1016/j.jbusres.2020.05.030 32457556 PMC7241379

[11] Bustamante–Ubilla MA. Chapter: Understanding Organizations from The Theories of Complexity and Social Ecology of Book: Man and his social environment, introduction, concept and perceptions, First Edition, 2015. University of Guanajuato and Superior Oversight Body of the Congress of the State of Guanajuato, Mexico. ISBN 978-607-441-335-9; 2015 [cited 2022 May 1]. https://www.researchgate.net/publication/301867206_Capitulo_ENTENDIENDO_LAS_ORGANIZACIONES_DESDE_LAS_TEORIAS_DE_LA_COMPLEJIDAD_Y_ECOLOGIA_SOCIAL

[12] BID, Banco interamericano de desarrollo. Pineda Emilio, Radics Axel. Collecting welfare. Subnational governments and coronavirus: five critical actions that we support from the IDB. 2020 [cited 2022 May 1]. https://blogs.iadb.org/gestion-fiscal/es/gobiernos-subnacionales-y-coronavirus-america-latina/

[13] Lee V, Chiew C. Interrupting transmission of COVID-19: lessons from containment efforts in Singapore. J Travel Med. 2020:1–5. doi: 10.1093/jtm/taaa039 32167146 PMC7107552

[14] Ministerio de hacienda. State of public finances. 2020 [cited 2022 May 1]. file:///C:/Users/Usuario/Downloads/EHP2020-WEB.pdf

[15] Solimano A, Pollack M, Wainer U, Wurgaft J. CIGLOB. 2007 [cited 2022 May 1]. https://www.ciglob.org/wp-content/uploads/2016/09/WP03-Solimano-Pollack-Wainer-Wurgaft-Micro-Empresas-PyMEs-y-Desarrollo-Econ.pdf

[16] Hambleton IR, Jeyaseelan SM, Murphy MM. COVID-19 in the Caribbean small island developing states: lessons learnt from extreme weather events. Lancet Glob Health. 2020; 8:e1114–5. doi: 10.1016/S2214-109X(20)30291-6 32622401 PMC7332258

[17] Umakanthan S, Lawrence S. Predictors of COVID-19 vaccine hesitancy in Germany: a cross-sectional, population-based study. Postgrad Med J. 2022. doi: 10.1136/postgradmedj-2021-141365 37062994

[18] Ministerio de Economía, Fomento y Turismo. Ministry of Economy, Development and Tourism. 2017 [cited 2022 May 1]. https://www.economia.gob.cl/wp-content/uploads/2017/03/Bolet%C3%ADn-empresas-en-Chile-ELE4.pdf

[19] Cancino C, La Paz A. International New Ventures en Chile: three success stories. Academy. Revista Latino Adm. 2010; 45:140–162. [cited 2022 May 1]. Available from: https://www.redalyc.org/articulo.oa?id=716/71615503010

[20] Ochoa SF, Robbes R, Marques M, Silvestre L, Quispe A. What Differentiates Chilean Niche Software Companies: Business Knowledge and Reputation. IEEE Software. 2017; 34:96–103. doi: 10.1109/MS.2017.64

[21] Cataldo A, Pino G, Mcqueen RJ. Size matters: the impact of combinations of ICT assets on the performance of Chilean micro, small and medium enterprises. Inf Tech Develop. 2020; 26:292–315.

[22] Antunes F, Ribeiro B, Pereira F. Probabilistic modeling and visualization for bankruptcy prediction. Appl. Soft Comput. 2017; 60:831–843. [cited 2022 May 1]. Available from: https://pdf.sciencedirectassets.com/272229/1-s2.0-S1568494617X00094/1-s2.0-S1568494617303861/main.pdf?X-Amz-Security-Token=IQoJb3JpZ2luX2VjEDoaCXVzLWVhc3QtMSJHMEUCIQDtGjTB7nofZ62sXzZoFuuf89N8VTROFc3ptoZUyR0g8gIgUPBcqpyR2lg9O6ZV0BU4mNFZop3xNfbUdRioJ%2BR8BP

[23] Umakanthan S, Bukelo MM, Gajula SS. The Commonwealth Caribbean COVID-19: Regions Resilient Pathway During Pandemic. Front. Public Health. 2022:844333. doi: 10.3389/fpubh.2022.844333 35664108 PMC9160791

[24] Caribbean Public Health Agency (CARPHA). CARPHA Situation Reports for COVID-19. 2021 [cited 2022 May 1]. https://carpha.org/Portals/0/Documents/COVID%20Situation%20Reports/Situation%20Report%20175%20-%20December%2015,%202021.pdf

[25] Luhmann N, De Giorgi R. Society Theory. 1ª. Ed. Guadalajara México: Universidad de Guadalajara/Universidad Iberoamericana/Iteso. 1993 [cited 2022 May 1]. http://bibliotecasibe.ecosur.mx/sibe/book/000020154

[26] Isac C, Guţă A. State Benefit—Incentive For Savings And Investments. Anna Univer Petroşani Econ. 2015; 15:63–70. [cited 2022 May 1]. Available from: https://search-proquest-com.utalca.idm.oclc.org/docview/2108802988?accountid=14675

[27] Rincón-González S, Mujica-Chirinos N. Evaluation from the perspective of the beneficiaries of the impact of the Barrio Adentro II Program in the state of Zulia. Espacios Públicos. 2014; 17:135–155. [cited 2022 May 1]. Availabe from: https://www.redalyc.org/articulo.oa?id=67635359007

[28] Instituto Regional de Administración de Empresas. XIV Business Perception Study. Concepción. 2017 [cited 2022 May 1]. https://irade.cl/wp-content/uploads/2014/10/Irade_2017.pdf

[29] Horta R, Camacho M, Silveira L, Ferreira L, Carnelli M. Business Perception Survey 2019. Montevideo. 2019 [cited 2022 May 1]. Available from: https://ucu.edu.uy/sites/default/files/facultad/fce/i_competitividad/encuesta-percepcion-empresarial-2018.pdf

[30] Seland D. Perception. Quality. 2016; 55:6. [cited 2022 May 1]. Available from: https://search-proquest-com.utalca.idm.oclc.org/docview/1788220190?accountid=14675

[31] Arias Castilla CA. Theoretical approaches on the perception that people have. Horiz Pedag, 2006; 8:9–22. [cited 2022 May 1]. Available from: https://horizontespedagogicos.ibero.edu.co/article/view/08101

[32] Kumar R. Perception! University Wire. 2017 [cited 2022 May 1]. https://search-proquest-com.utalca.idm.oclc.org/docview/1882150911?accountid=14675

[33] Bustamante-Ubilla MA, Del Río-Rivero MC, Lobos-Andrade GE, Villarreal-Navarrete PI. Perception of the motivation of middle managers in three hospitals in the Maule Region, Chile. Public Health of Mexico. 2009; 51:417–426. [cited 2022 May 1]. Available from: http://www.scielo.org.mx/scielo.php?script=sci_arttext&pid=S0036-36342009000500009&lng=es&tlng=es 10.1590/s0036-36342009000500009 19936555

[34] Senge P. The Firth Discipline Fierldook: Strategies and tools for building a learning organization. Editions Nicholas Bready, London; 1994.

[35] Luhmann Niklas. Society and system: the ambition of the theory. Editorial Paidos, Barcelona, España, p.23. 1997 [cited 2022 May 1]. https://dialnet.unirioja.es/servlet/libro?codigo=232297

[36] Hernández-Sampieri R, Mendoza C. Research methodology: The quantitative, qualitative and mixed routes, Mexico City, Mexico: Editorial Mc Graw Hill Education, ISBN: 978-1-4562-6096-5, 714. 2018 [cited 2022 May 1]. http://virtual.cuautitlan.unam.mx/rudics/?p=2612

[37] Lorenzo-Seva U, Ferrando PJ. FACTOR 9.2: A comprehensive program for fitting exploratory and semiconfirmatory factor analysis and IRT models. Appl Psychol Meas. 2013; 37:497–498. doi: 10.1177/0146621613487794

[38] Marsh HW, Morin AJS, Parker PD, Kaur G. Exploratory Structural Equation Modeling: An Integration of the Best Features of Ex-ploratory and Confirmatory Factor Analysis. Annu Rev Clin Psychol. 2014; 10:85–110. doi: 10.1146/annurev-clinpsy-032813-153700 24313568

[39] Freiberg HA, Stover JB, de la Iglesia G, M Fernández L. Polychoric and Tetrachoric Correlations in Exploratory and Confirmatory Factorial Studies. Prensa Médica Latinoamericana. Ciencias Psicológicas. 2013; 7:151–164 [cited 2022 May 1]. Available from: https://www.redalyc.org/pdf/4595/459545415005.pdf

[40] SII. Department of Economic and Tax Studies of the Strategic Management and Tax Studies Subdirectorate of the Internal Revenue Service. 2020 [cited 2022 May 1]. https://www.sii.cl/sobre_el_sii/nominapersonasjuridicas.html

[41] IM Talca. Ilustre Municipalidad de Talca. Active commercial patents during the first quarter of 2020, issued by the Headquarters of the Transparency Unit of the Illustrious Municipality of Talca. 2020 [cited 2022 May 1]. https://www.portaltransparencia.cl/PortalPdT/directorio-de-organismos-regulados/?org=MU312

[42] Sampieri RH. Investigation methodology. Mexico D.F.: McGRAW-HILL. 2010.

[43] Cañadas IO, Sánchez AB. Response Categories On Likert-Type Scales. Psicothema. 1998; 10:623–631 [cited 2022 May 1]. Available from: http://www.psicothema.com/psicothema.asp?id=191

[44] Cabero-Almerara J, Del Prete A, Arancibia Muñoz ML. Perceptions of Chilean university students about the use of social networks and collaborative work. Rev Iberoame Edu Distan. 2019; 22:35–55 [cited 2022 May 1]. Available from: http://revistas.uned.es/index.php/ried/article/view/22847/19879

[45] Ferrando PJ, Anguiano-Carrasco C. Factor Analysis As A Research Technique In Psychology. Papeles Psicól. 2010; 31:18–33. [cited 2022 May 1]. Available from: https://www.redalyc.org/articulo.oa?id=778/77812441003

[46] Lloret-Segura S, Ferreres-Traver A, Hernández-Baeza A, Tomás-Marco I. The exploratory factor analysis of the items: a practical guide, revised and updated. Ann Psychol. 2014; 30:1151–1169.

[47] Garmendia ML. Factor analysis: an application on the Goldberg General Health Questionnaire, 12-question version. Rev Chilena Salud Públ. 2007; 11:57–65 [cited 2022 May 1]. Available from: https://revistasaludpublica.uchile.cl/index.php/RCSP/article/view/3095/2963

[48] Pérez E, Medrano L. Exploratory factor analysis: Conceptual and methodological bases. Rev Argentina Cien Comport. 2010; 2:58–66 [cited 2022 May 1]. Available from: https://dialnet.unirioja.es/servlet/articulo?codigo=3161108

[49] Guarín A, Ramírez A, Torres F. Modelos Multinomiales: Un Análisis De Sus Propiedades. Revista Ingenierías Universidad de Medellín. 2012; 11:87–104 [cited 2022 May 1]. Available from: https://ideas.repec.org/p/col/000122/010604.html

[50] Chión S, Charles V. Analytics for Structural Modeling. Perú. Pearson Perú; 2016;239–322.

[51] Escobedo Portillo MT, Hernández Gómez JA, Estebané Ortega V, Martínez Moreno G. Structural equation models: Characteristics, phases, construction, application and results. Ciencia & Trabajo. 2016; 18:16–22.

[52] Forero CG, Maydeu-Olivares A, Gallardo-Pujol D. Factor Analysis with Ordinal Indicators: A monte Carlo study comparing DWLS and ULS estimation. Struct Equ Modeling. 2009; 16:625–641. doi: 10.1080/10705510903203573

[53] Beavers AS, Lounsbury JW, Richards JK, Huck SW, Skolits GJ, Esquivel SL. Practical considerations for using exploratory factor analysis in educational research. *Pract*. *Assess*. *Res*. *Evaluation*. 2013; 18:6 [cited 2022 May 1]. Available from: https://scholarworks.umass.edu/cgi/viewcontent.cgi?article=1303&context=pare

[54] Ministerio de Salud. COVID-19 in Chile, Pandemic 2020–2022. 2022 [cited 2022 May 1]. https://www.minsal.cl/wp-content/uploads/2022/03/2022.03.03_LIBRO-COVID-19-EN-CHILE-1-1.pdf

[55] Benedettini O, Swink M, Neely A. Examining the influence of service additions on manufacturing firms’ bankruptcy likelihood. Ind Mark Manag. 2017; 60: 112–125. doi: 10.1016/j.indmarman.2016.04.011

[56] Crespi G. SMEs in Chile: born, grows and… dies. Analysis of its development in the last seven years. *FUNDES*. 2003 [cited 2022 May 1]. Available from: https://coyunturapolitica.files.wordpress.com/2008/07/pyme-en-chile-nace-crece-y-muere-informe-2003.pdf

[57] Lopucki LM, Doherty JW. Bankruptcy Survival. UCLA Law Review. 2015; 62:970–1015 [cited 2022 May 1]. Available from: http://search.ebscohost.com.utalca.idm.oclc.org/login.aspx?direct=true&db=bsu&AN=103536021&lang=es&site=ehost-live

[58] Barboza F, Kimura H, Altman E. Machine learning models and bankruptcy prediction. Exp Syst. Appl. 2017; 83:405–417. doi: 10.1016/j.eswa.2017.04.006

[59] Alaminos D, del Castillo A, Fernández MÁ. A Global Model for Bankruptcy Prediction. PLOS ONE. 2016; 11:e0166693. doi: 10.1371/journal.pone.0166693 27880810 PMC5120822

[60] Arroyave J. A comparative analysis of the effectiveness of corporate bankruptcy prediction models based on financial ratios: Evidence from Colombia. J Int Stud. 2018; 11:273–287.

[61] Yoguel G, Barletta F, Pereira M. From Schumpeter to the post-Schumpeterians: old and new analytical dimensions. Rev Probl Des. 2013:174 [cited 2022 May 1]. Available from: https://www.google.cl/url?sa=t&rct=j&q=&esrc=s&source=web&cd=&ved=2ahUKEwikqazc_6PsAhUrD7kGHT06C6oQFjAAegQIBhAC&url=http%3A%2F%2Fwww.revistas.unam.mx%2Findex.php%2Fpde%2Farticle%2Fdownload%2F40289%2F36668&usg=AOvVaw2wKOh0fYht1FNmA6njwI1d

