# Supplementary material for: Perception study of state support programs for companies, in Talca, Chile in times of Covid 19
Source: PLoS One. 2022 Oct 7;17(10):e0274051. doi: 10.1371/journal.pone.0274051 (PMC9543626; doi:10.1371/journal.pone.0274051)
Supplement: S2 File — (DOCX) [file pone.0274051.s002.docx]

**QUESTIONNAIRE**

**Perception Study of State Support Programs for companies, Talca, Chile**

**in times of Covid 19**

Dear

The Escuela Superior Politécnica del Litoral ESPOL of Ecuador is carrying out a study entitled: "Study of Perception of State Support Programs for companies, Talca, Chile in times of Covid 19". The objective of this study is to identify the factors that affect the perception that Talca companies have of state programs aimed at helping organizations in times of COVID 19. Your authorization is requested to participate in this research project whose objective is study ecotourism to develop the destination. Your participation is completely voluntary, if you do not wish to participate, there will be no negative consequences. You can withdraw from the study at any time. The response is completely anonymous. There is no associated risk. If you have any questions, you can contact Wilmer Carvache -Franco whose e-mail is: wcarvach@espol.edu.ec

If I agree to participate ❑

I do not agree to participate ❑

**First part**

Below is a series of questions aimed at knowing the profile of your company. In the rows below, you are asked to select the option that best represents your company identification.

1. How many years has your company been in the market?

| 0 - 4 years ___ | 5 – 9 years ___ | 10 – 14 years ___ | 15 or older ___ |
| --- | --- | --- | --- |

1. How many employees does your company currently have?

| 0 – 9 employees ___ | 10 -24 employees ___ | 25 – 199 employees___ | 200 or more employees ___ |
| --- | --- | --- | --- |
|  |  |  |  |

1. Has your company applied to any government program?

| YES ___ | NOT ___ |
| --- | --- |

1. Has your company been beneficiaries of government programs?

| YES ___ | NOT ___ |
| --- | --- |

1. Select the name of the government institution from which you have received benefits. If your company has not been a beneficiary, select the option “ **NOT APPLICABLE** ”.

| **Institution** | **YES** | **DOES NOT APPLY** | **Institution** | **YES** | **DOES NOT APPLY** |
| --- | --- | --- | --- | --- | --- |
| **CORFO** |  |  | **SENAMA** |  |  |
| **SERCOTEC** |  |  | **INDAP** |  |  |
| **PHOSIS** |  |  | **PROCHILE** |  |  |
| **FIA** |  |  | **SENCE** |  |  |
| **SERNAMEG** |  |  | **INAPI** |  |  |
| **SERNATUR** |  |  | **SENADIS** |  |  |
| **INJUV** |  |  |  |  |  |

Second part

Below is a series of statements regarding government benefits aimed at companies. In the rows below, you are asked to rate the statements by marking your preference, selecting the answer that best represents your opinion.

Below is the table of scores.

| one | two | 3 | 4 | 5 |
| --- | --- | --- | --- | --- |
| *strongly disagree* | ***In disagreement*** | ***Indifferent*** | ***Okay*** | ***Totally agree*** |

| Competitiveness Area | one | two | 3 | 4 | 5 |
| --- | --- | --- | --- | --- | --- |
| 1. Do you think that benefits allow a company to be more competitive? |  |  |  |  |  |
| 1. Do you think that if your company obtains benefits, it will be able to consolidate itself in the market? |  |  |  |  |  |
| 1. Do you think that if companies receive benefits, this will bring about an increase in market competitiveness? |  |  |  |  |  |
| Area Barriers to entry | one | two | 3 | 4 | 5 |
| 1. Do you think that if a new company receives benefits, the obstacles of the market decrease? |  |  |  |  |  |
| 1. Do you think that if a company with a track record receives benefits, market obstacles increase? |  |  |  |  |  |
| 1. Do you think that if a new company does not make a profit, it will see an increase in market barriers? |  |  |  |  |  |
| Financial Area | one | two | 3 | 4 | 5 |
| 1. Do you think that the economic benefits should vary according to the size of the company? |  |  |  |  |  |
| 1. Do you think that the economic benefits should vary according to the market in which the company is located? |  |  |  |  |  |
| 1. Do you think that the economic benefits should vary according to the current financial situation of the company? |  |  |  |  |  |
| 1. Do you think that if your company receives economic benefits, it will be able to decide not to opt for financial credits? |  |  |  |  |  |
| Productivity Area | one | two | 3 | 4 | 5 |
| 1. Do you think that the training programs allow to increase the performance of the employees? |  |  |  |  |  |
| 1. Do you think that access to government benefits will allow your company to develop more efficient processes? |  |  |  |  |  |
| 1. Do you think that receiving training will allow your company to develop greater production at a lower cost? |  |  |  |  |  |
| Investment Area | one | two | 3 | 4 | 5 |
| 1. Do you think that if your company receives money from the state, with it you can increase remuneration? |  |  |  |  |  |
| 1. Do you think that if your company receives money from the state, it could be invested in infrastructure? |  |  |  |  |  |
| 1. Do you think that if your company receives money from the state, it can be used to design new products and services? |  |  |  |  |  |
| Employee Knowledge and Relationship Area | one | two | 3 | 4 | 5 |
| 1. Do you think that if you receive training you will be able to increase your knowledge in the area in which you work? |  |  |  |  |  |
| 1. Do you think that if a worker receives training he will be able to generate ideas that contribute to the development of the company? |  |  |  |  |  |
| 1. Do you think that if your company receives benefits, it could improve the working conditions of its employees? |  |  |  |  |  |

Third part

Next, a series of affirmations are presented regarding general aspects in; allocation, application and obtaining government benefits. In the rows below you are asked to evaluate each of these, selecting the answer that best represents your opinion. Below is the table of scores.

| one | two | 3 | 4 | 5 |
| --- | --- | --- | --- | --- |
| *strongly disagree* | ***In disagreement*** | ***Indifferent*** | ***Okay*** | ***Totally agree*** |

|  | one | two | 3 | 4 | 5 |
| --- | --- | --- | --- | --- | --- |
| 1. Do you think that the age of a company determines whether or not it has access to government benefits? |  |  |  |  |  |
| 1. Do you think that the benefits are currently accessible to all types of companies? |  |  |  |  |  |
| 1. Do you think that the benefits should be directed to regions with high unemployment rates? |  |  |  |  |  |
| 1. Do you think that if the requirements for benefits are reduced, would the application for them increase? |  |  |  |  |  |
| 1. Do you think there is a need to improve the promotion that benefits currently have? |  |  |  |  |  |
